# 

**Published:** 1856-01

**Authors:** 


### INDEX FOR VOLUME III.

Agriculture.................. 23, 140
Aristocracy of Blood............ 150
Air In and Out Door............. 199
Bath Rooms............... ..... 91
Bathing ....................... 269
Barnum's Failure............ ... 78
Bread. Healthy.................. 144
Bad Colds.... ................. 192
Brandy Drinking................ 254
Bronchitis...................... 207
Buckwheat Cakes................ 267
Consumption........... 8,182,205,229
Corns.......................... 22
Clerical Mortuary................ 68
Civilization, True............... 94
Church Sleeping ................ 95
Church Leaving.................. 118
Checked Perspiration. ........... 121
Cough Remedy.............. 123,184
Clerical Recreation.............. 128
Children's Health................ 149
Count Confalioneri............... 153
Cinders in the Lye............... 198
Christmas Happified.............. 270
Clerical Employments............ 276
Disease and Providence.......... 1
Domestic Receipts............... 47
Death, Cause of................. 85
Dress.......................... 98
Dead of a Dinner................ 114
Donation Parties ................ 138
Damp Walls................. ... 160
Daughters....................... 245
Dietetics ........... .......... 250
Disease Foreshadowed............ 263
"Dred" Noticed................. 275
Equanimity of Mind............. 15
Editors............. 31,39,97,126,156
Exercise................... 112,147
Early Marriages.................. 153
Eyes.................... 198,208,158
Easy Circumstances.............. 195
Felt Hats ...................... 95
Food .................. 10,157,250
Forgetting......... ............ 274
Family Peace.................... 119
Feet, Care of.................. 133
Fruits ......................... 154
Filth, Reproductive.............. 201
Godey's Lady's Book ........... 140
Hall, Alice ..................... 275
Health Seeking........13,56,70,93,115
Hard Study...................... 42
Hair Dye ....................... 99
Healthy Bread................... 144
Hats, Best...................... 95
Hereditary Disease .............. 191
Ideal Value..................... 270
Inhalation .................. 156,226
Insanity...................... 53,224
Influenza .................. 1,30,139
Irritabilities of Life............. 209
Instructive Narrative......... 204,252
Keep Mouth Shut................ 21
Long Life ................. 204,252
Life Lost....................... 258
Memory Improved............... 262
Mental Health ............. 15,87,111
Medical Phantasies .............. 36
Money Lending .................. 84
Model Minister.................. 101
MacFarland, John................ 101
Medicine, Our .................. 120
Mushrooms...................... 200
Marriage, Early............. 153,200
Memory Improved............... 24
Morals of the Press............ 31,72
Milk, About..................... 274
Out-Door Activities.............. 234
Newspaper Patronage............ 264
Nervous Diseases................ 272
Night Air....................... 142
Providence and Disease........... 1
Poverty......................... 7
Practical Knowledge............. 19
Physical Education.............. 253
Physician, The Good............. 263
Proclivities of the Age........... 12
Preserved Sunshine.............. 33
Physiognomy.................... 41
Presentiments.................. . 44
Physic and Politics.............. 61
Patent Medicines................ 63
Piles, Cause of.................. 66
Polar Sea, Health in............. 70
Popular Fallacies............. 141,77
Peace in the Family............. 119
Poisonous Mushrooms............ 120
Pulse ..................... 229,291
Perspiration .................... 248

Quo Modo.......................... 63
Quackery.......................... 130
Religious Press.................. 31
Review Morals.................... 72
Railroad Safety.................. 75
Receipts, Domestic.... ... 47,100,204
Rules for Sick-Room............ 202
Respirators...................... 265
Seeking Health................... 13
Sick Mind........................ 16
Sunday Dinners................... 34
Sleeplessness.................... 46
Spiritualism..................... 88
Sleeping in Church............ 95,156
Sense and Nonsense............. 112
Sea Voyages......................
Sleep of Nature.................. 166
Sick Room Rules ................. 202
Spitting Blood .................. 209
Southern Climate................. 249
Small Pox. ...... ............... 271
Sharpe, Ebenezer................. 271
Schooling Errors................. 275
Suicide.................. ...... 259
Simple Medicines................. 260
Sleep, Sound.............. ...... 261
Thermometers, Uses of........... 119
Tooth Wash....................... 228
Throat Ail.................. 135,206
Tight Lacing..................... 188
Temperance................. 203,246
Tubercle Described.......... 208,214
Theological Studies.............. 272
Unhealthy Bread.................. 47
Use of Fruits.................... 155
Why?............................. 11
Wives.................... 41,119,247
Weak Eyes........................ 203
Wisdom of the Wicked............. 203

### TO OUR EXCHANGES.

We feel much indebted for the favorable notices which they have uniformly taken of our *Journal*, as well as for the compliment implied in so frequently extracting from its pages; excepting the leaders, and repeatedly, even them, nearly every article of each number is thought worthy of being copied by some one paper or other. Single papers have several times taken more than a yard of our matter for one issue. Our aim for the present year is to write more worthy of quotation, to write usefully, practically, courteously. We venture to ask of our Exchanges an increase of our obligations to them, by their giving credit to such articles as they may hereafter think worthy of being copied, as from Hall's N. Y. Journal of Health, for the reason, that we receive letters from time to time, stating that long ago, the writers would have *subscribed*, had they been able to ascertain where the *Journal* was published, their attention having been drawn to it, by the frequent extracts from our pages; so, while obliging us, our Exchanges will at the same time impart desired information to their own subscribers.

We will hereafter send our Journal to any name desired by an exchanging Editor, for Fifty Cents a year, always beginning with the January number of the current year; *provided* such name is a *paying* subscriber to the paper of said Editor—in other *words*, the *club* system.

We send this January number to no one subscriber of last year, unless to such as have sent their dollar. On our list we have the names of eminent men of the time, lawyers, clergymen, physicians; rich men, who count their fortunes by hundreds of thousands and by millions, but the prompt dollar of a mechanic is worth to us more than the tardy dollar of the millionaire. We offer no premium to the indifferent and the unmethodical. A man who is not prompt enough to pay a dollar at the beginning of a year, is not likely to pay it at the end of a year without a reminder. To one of our sensibilities, it is worth a dollar to ask for it:—we have no idea of earning a dollar in the first place, and earning it a second time in sending or writing for it. We do not publish for a dollar, but for a *prompt dollar!*

---

### NOTICE TO SUBSCRIBERS.

THE reception of two successive numbers of this *Journal* is evidence that it is paid for, for the current year.

All Subscriptions begin with January of the current year; being stereotyped, back numbers can be furnished to any desired extent.

Postage is THREE CENTS a year in the State of New York; *Six Cents* a year out of it, payable in advance, where it is received.

Our City Subscribers will have it furnished at their doors for $1 15cts. a year. Those paying $1, will receive it through the General Post-Office.

Volumes ONE and TWO of *Hall's Journal of Health*, for 1854

and 1855, with copious Alphabetical Index, bound uniformly in cloth, are sold at the Editor's office, for $1 25cts. each; and will be sent post-paid for ten cents each, additional. As we confine our teachings about health to the habits and practices of life, recommending no medicine, detailing no symptoms, employing no professional terms, it is the frequently expressed opinion of the newspaper press, that this Journal merits a place in every family, and that it will be useful for reference in after life, as well as to-day.

---

### NOTICES OF BOOKS, PERIODICALS, &C.

*Scenes in the Practice of a New York Surgeon*, 407 pp. 12mo, finely got up, with interesting illustrations byDewitt & Davenport, by Edward H. Dixon, M. D., a keen writer, a medical scholar and a talented man. The book is like him, and imparts a large amount of information, interesting, truthful and practical. It is sure of a large sale.

Musical World. A Literary and Fine Art Paper, by Richard Storrs Willis, 257 Broadway, New York, 16 pp. 4to, 3 columns each, published weekly at Two Dollars a year, only *if paid in advance.* It is now in its 13th vol. and has been edited with industry, ability, and great good taste. Four pages of selected Music are given each week, paged separately, and well bound at the end of the year, will form a volume of music, which would cost Twelve Dollars, if purchased in ordinary sheet form. A Musical Novelette will be commenced with January, besides a variety of instructive musical reading, articles on vocal and instrumental culture, with a new department of *general Literature.* We believe that every boy and girl should be taught to sing. The knowledge of music, aside from its practice, is elevating and refining, not only to the performer, but to the hearer, and the neglect of its cultivation is a serious defect; it is a national oversight and is inexcusable. There can be no crime within the hearing of pure music; it exorcises devilism now, as it did in King David's time. The natural vent of happiness is song, and well may we imagine it to be the frequent employment of angels, and the spirits of just men made perfect in Heaven.

Banner of the Cross, Philadelphia. An Episcopal Weekly, at $2 50 a year, in advance. Edited by John Coleman, D. D., is welcome to our exchange list. It is filled with *short* articles of news, and practical items; *not* made heavy as lead, by whole columns of *Foreign Correspondence* of Tom, Dick and Harry, and whoever wants to see his scribblings in print, and will scribble for nothing. These same scribblings, paid for or not, practically, take the place, *as religious reading*, of the Bible on Sundays. We believe that the reading of so-called "Religious Papers," is, in most instances, a desecration of the Sabbath Day. No wonder all the religious denominations are lamenting the coldness of the churches; they will continue to lament that coldness, until the Bible, for Sunday reading and meditation, supplants the "Religious Newspapers." as now generally conducted, as to spirit, manner and matter.

The Christian Review, Quarterly, James J. Woolsey, 115 Nassau street, New York, is well worthy of the patronage of an enlightened public. Three Dollars a year.

